# Efficacy and Safety of Botulinum Toxin A and Pulsed Radiofrequency on Postherpetic Neuralgia: A Randomized Clinical Trial

**DOI:** 10.1155/2022/1579937

**Published:** 2022-05-30

**Authors:** Liping Chen, Yaoyao Zhang, Yanru Chen, Ting Wang, Kai Sun, Hao Tang, Wen Shen, Fuhai Ji

**Affiliations:** ^1^The First Affiliated Hospital of Soochow University Department of Anesthesiology, Suzhou 215006, Jiangsu, China; ^2^The Affiliated Hospital of Xuzhou Medical University Department of Pain, Xuzhou 221000, Jiangsu, China; ^3^Xuzhou Medical University Institute of Anesthesia, Xuzhou 221000, Jiangsu, China; ^4^The Second Affiliated Hospital of Xuzhou Medical University Department of Pain, Xuzhou 221002, Jiangsu, China

## Abstract

This study evaluated the effectiveness and safety of botulinum toxin type A (BoNT-A) and pulsed radiofrequency (RF) in the clinical treatment of postherpetic neuralgia (PHN). A total of 100 patients with PHN were randomly divided into two groups (*n* = 50 per group): RF group and BoNT-A group. Based on conventional drug treatment, patients were treated with either a single nerve root pulsed radiofrequency therapy or a single local subcutaneous injection of BoNT-A in the lesion area. All the patients were followed up for 24 weeks on pain scores, sleep quality, anxiety, and depression scores, etc. In the last follow-up at the end of 24 weeks postoperation, the pain scores of patients in both groups were significantly lower than those before the operation (*P* < 0.05), indicating that both treatments were effective against PHN; however, there was no significant difference between these two groups (*P* > 0.05). It is noteworthy that the subcutaneous injection of BoNT-A is relatively easy to administer and less expensive compared to RF. Therefore, we believe that the subcutaneous injection of BoNT-A is an effective and safe method for the treatment of PHN.

## 1. Introduction

Shingles is caused by the reactivation of latent varicella-zoster virus (VZV) infection in the primary sensory neurons of the human body. Once reactivated, the VZV spreads along the sensory nerves and causes herpes in the corresponding cutaneous segment, which is often accompanied by severe pain [1]. In most patients, the pain is supposed to be gradually improved with the rash heals. However, about 9%–34% of patients with shingles still suffer from pain that persists beyond the rash has healed [2]. To distinguish with the acute phase of herpes zoster, the period from 30 to 90 days after the rash onset is often referred to as the subacute phase of herpes zoster, and the pain presents for 90 days or more after rash onset is defined as postherpetic neuralgia (PHN) [3–5]. With the development of understanding of the disease, PHN has been defined clinically as pain that lasts more than 1 month after the complete healing of the rash [1–6]. Studies have shown that PHN is a typical neuropathic pain, often prolonged and seriously affecting the life and work of patients and their families. PHN not only causes long-term severe pain in the lesion area, but also causes sleep disorders and anxiety, which seriously reduces the quality of life of patients, and some patients even have depression and suicidal tendencies [7, 8].

Since Sluijter first proposed pulsed radiofrequency technology at the second Annual Conference of the European Federation of International Pain Societies in 1997, pulsed radiofrequency technology has been widely used in pain treatment due to its advantages of convenient operation, safety and effectiveness, minimally invasive, and fewer complications. The mechanism of pulsed radiofrequency technology for pain relief is mainly electric field effect; that is, pulsed radiofrequency technology generates voltage fluctuation between tissues requiring treatment through high-frequency current, thus generating periodic electric field activity and regulating synapses to achieve pain relief [8]. Evidence-based evaluation and analysis have shown that pulsed radiofrequency technology can relieve the symptoms of neuralgia after zoster, significantly reducing pain within 2–3 days, and the effect lasts for six months [9].

Botulinum toxin type A (BoNT-A) is one of the natural toxins produced by Clostridium botulinum. Clinically, it is mainly used for the treatment of cosmetology and spasmodic diseases, including hemifacial spasm, spasmodic torticollis, and neurogenic bladder. [10–12]. However, both animal and clinical studies have suggested that BoNT-A is effective in the treatment of neuropathic pain [13–15], including PHN [16, 17], when applied placebo as a control. The direct comparison between BoNT-A and other effective treatments remains largely overlooked. A meta-analysis conducted by Li et al. [18] indicated that the subcutaneous injection of BoNT-A could effectively treat PHN with advantages of high safety, few adverse reactions, simple treatment, and economic application.

Through a randomized, single-blind, parallel-controlled clinical research, we compared the clinical efficacy and safety of pulsed radiofrequency (RF) and subcutaneous injection of BoNT-A in the treatment of PHN, observed the sensitivity of different characteristics of PHN to these two treatments, and further optimized the clinical strategy for the treatment of PHN.

## 2. Methods

### 2.1. Subjects

This study was registered in the Chinese Clinical Trial Registry. The registration number is ChiCTR1900020798. The research content was approved by the Ethics Committee of the Affiliated Hospital of Xuzhou Medical University (protocol code: XYFY2019-KL046-02) and strictly followed the Declaration of Helsinki. The whole process was completed in the Affiliated Hospital of Xuzhou Medical University. The study started on December 9, 2019, and the last patient completed the last follow-up on February 24, 2021.

The inclusion criteria for this study were as follows: (1) pain lasted for more than one month after the herpes zoster was completely healed, (2) ID-Pain scale ≥2, (3) age ≥18 years, and (4) NRS ≥4.

**Figure 1:**
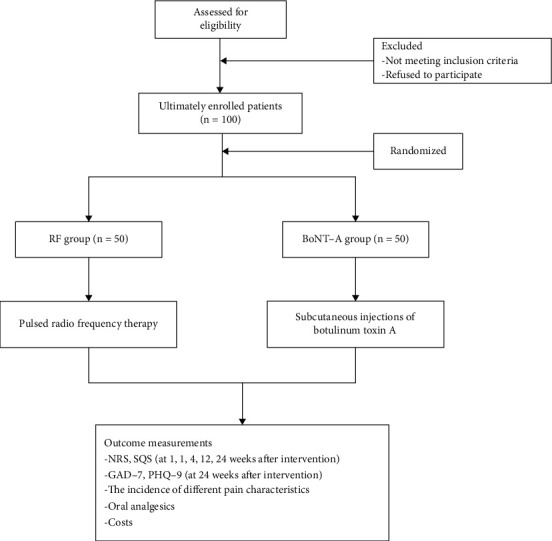
Study flow diagram.

The exclusion criteria were as follows: (1) there was a history or family history of neuromuscular junction disease; (2) drugs acting on the neuromuscular junction or aminoglycoside antibiotics had been used in the past 2 weeks; (3) there was a personal or family history of coagulation dysfunction; (4) BoNT-A was used in the recent 6 months; (5) there were severe systemic diseases (e.g., severe heart failure, liver, and renal failure); (6) women who were preparing for pregnancy, in the pregnancy or lactation period; (7) there were other conditions that would affect pain assessment (e.g., vertebral compression fracture and multiple myeloma); (8) allergy to botulinum toxin A, lidocaine, gabapentin, tramadol, or oxycodone; (9) patients were unable to communicate, or with severe mental disorder, alcohol addiction, or drug abuse; and (10) patients refused the treatments. The study flow diagram is shown in Figure 1.

### 2.2. Randomization and Blindness

The computer software (https://random-allocation-software.software.informer.com/2.0/) was used to conform to the condition that 100 patients were randomly assigned according to 1 : 1. Patients and surgeons knew about the grouping, but the follow-up personnel and statisticians did not. Follow-up did not involve the specific situation of grouping.

### 2.3. Treatments

Baseline information collection and preoperative assessment were completed on the day before treatment, and then, patients were randomized and treated, with four follow-up visits at the weekends 1, 4, 12, and 24 after treatment. Throughout the follow-up, the patients were able to adjust the dose of oral analgesics, which were mainly gabapentin, tramadol, or oxycodone according to the patients' pain level.

The procedure for BoNT-A injection was as follows: referring to previous injection methods [12, 13], the patients were lying supine or sideways, labeled according to the range of pain reported. The surgical field was routinely disinfected. 100 units of BoNT-A (Heng Li, Lanzhou Biological Products Research Co., Ltd.) were diluted with 4 ml saline (25 units/ml) and injected subcutaneously within the labeled area at an interval of 1.5–2 cm (0.1 ml for each site, i.e., 2.5 units of BoNT-A). The total dose was determined by the area of pain and did not exceed 80 points (i.e., the maximum dose was 200 units).

The procedure for pulsed radiofrequency was as follows: the patients lay prone, and the corresponding spinal segment was localized and marked by ultrasound according to the range of pain reported. The surgical field was routinely disinfected. A 22 G radiofrequency trocar (Inomed Inc., USA) was inserted into the plane along the lateral edge of the ultrasound probe and stopped when the tip reached the paravertebral space. The needle core was removed, and the radiofrequency electrode (Cosman, Boston Scientific Corporation, USA) was inserted. Then, the radiofrequency pain therapy instrument (RFG-4, Cosman, Boston Scientific Corporation, USA) was connected to give sensory stimulation of 0.1–0.3 mV at 50 Hz, which could induce the presence of abnormal sensation in the corresponding nerve distribution area. Subsequently, standard pulsed radiofrequency treatment was given: temperature 42 °C, frequency 2 Hz, pulse width 20 ms, voltage 37–41 V, and time 360 s.

The main outcome measure of this study was the NRS of the two groups before and after surgery (including baseline, 1 week, 4 weeks, 12 weeks, and 24 weeks after surgery).

Secondary outcome measures included the following: (1) sleep quality scores (SQSs) before and after surgery (including baseline, 1 week, 4 weeks, 12 weeks, 24 weeks after surgery); (2) GAD-7 and PHQ-9 scores before and 24 weeks after surgery; (3) proportion of different pain characteristics based on the ID-Pain scale before and 24 weeks after surgery; (4) oral analgesics at 24 weeks after surgery; (5) costs associated with treatments; and (6) occurrence of adverse events.

### 2.4. Statistical Analysis

According to the method of estimating the sample size of the noninferiority test and the results of previous research studies [19, 20], the mean NRS at 24 weeks after pulsed radiofrequency was about 3.0, and so 1/5 of 3.0 was selected as the noninferiority threshold *δ*, that was, 0.6. Assuming that the standard deviation between the two sets of data was approximately equal, we selected *α* = 0.025 (one-sided) and 1 − *β* = 0.8. *N*1 = *N*2 = 45 was calculated by the PASS 11. Taking into account the 10% dropout rate, 50 patients were proposed for each group.

Statistical software IBM SPSS 24.0 was used for statistical analysis of the data. The MICE program package in R software was used for multiple data filling. GraphPad Prism 5.0 was used for mapping. The Shapiro–Wilk test was used to determine the normality of the data distribution, and the Levene method was used to test the homogeneity of variance. The measurement data satisfying the normal distribution were represented by mean ± standard deviation $\bar{\text{X}}\pm \text{S}$, and those that did not meet the normal distribution were represented by median (M) and interquartile range (IQR). When the data were normally distributed and the variance was homogeneous, two independent sample *t*-test was used for comparison between groups, and repeated measures ANOVA was used at different time points within the groups. The Mann–Whitney *U* test was used for non-normally distributed data. The chi-square test or Fisher's exact probability test was used for count data, and the rank sum test was used for rank data. Two-factor repeated measures ANOVA or generalized estimating equation (GEE) was used for comparison between two groups at multiple time points. *P* < 0.05 was considered to be statistically significant.

## 3. Results

According to the inclusion and exclusion criteria, 100 patients voluntarily participated in this study that started on December 10, 2019, and the last patient completed the last follow-up on May 19, 2021. All patients were randomized into two groups (*n* = 50): RF group and BoNT-A group. The basic information of the two groups is shown in Table 1.

### 3.1. Subjective Pain

A numerical rating scale (NRS) was used to score the pain of all patients at baseline and 1 week, 4 weeks, 12 weeks, and 24 weeks postoperation. 0 represents no pain, and 10 represents the most severe pain. The subjects selected one number from the 11 numbers ranging from 0 to 10 to represent the degree of pain based on their personal subjective pain perception. The larger the number, the more severe the pain; the smaller the number, the less the pain.

**Figure 2:**
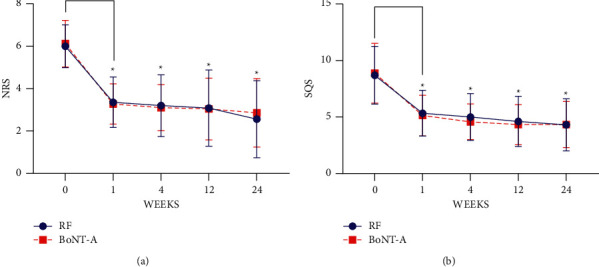
NRS and SQS scores of patients in both groups at baseline and 1 week, 4 weeks, 12 weeks, and 24 weeks postoperation. ^*∗*^=*P* < 0.05 vs. baseline.

There was no statistical difference in the baseline NRS between the two groups (*P* > 0.05). The NRS at 1 week, 4 weeks, 12 weeks, and 24 weeks postoperation were significantly lower than the baseline value (*P* < 0.05). However, at the same point in time, there was no significant difference in NRS between the two groups (*P* > 0.05), as shown in Figure 2(a).

### 3.2. Quality of Sleep

A score sheet containing five questionnaires [16] was used to score the sleep quality of all patients at baseline and 1 week, 4 weeks, 12 weeks, and 24 weeks postoperation, as shown in Table 2.

There was no statistical difference in the baseline sleep quality scores (SQS) between the two groups (*P* > 0.05). The SQS at 1 week, 4 weeks, 12 weeks, and 24 weeks postoperation were significantly lower than the baseline value (*P* < 0.05). However, at the same point in time, there was no significant difference in the scores between the two groups (*P* > 0.05), as shown in Figure 2(b).

### 3.3. Anxiety and Depression Scores

There is a significant correlation between pain, sleep, and mood. Pain may cause anxiety and/or depression in patients, and vice versa [21]. Therefore, generalized anxiety disorder 7-item (GAD-7) and patient health questionnaire-9 (PHQ-9) were used to observe the effects of the two treatments on the emotional state of patients with PHN.

There was no statistical difference in GAD-7 scores between the two groups at baseline (*P* > 0.05). At 24 weeks postoperation, GAD-7 scores in the RF group were significantly lower than those at baseline (*P* < 0.05). Although the GAD-7 scores in the BoNT-A group were also lower than that at baseline, the difference was not statistically significant (*P* > 0.05). The postoperative GAD-7 scores in the BoNT-A group were lower than that in the RF group, and the difference was statistically significant (*P* < 0.05), as shown in Table 3.

There was no statistical difference in PHQ-9 scores between the two groups at baseline (*P* > 0.05). At 24 weeks postoperation, PHQ-9 scores of the two groups were lower than those at baseline (*P* < 0.05), but there was no significant difference between the two groups (*P* > 0.05), as shown in Table 4.

### 3.4. Characteristics Based on ID-Pain

The ID-Pain scale contains 5 different pain characteristics of neuropathic pain: stabbing pain, burning pain, numbness, electric shock pain, and allodynia. And spontaneous pain often plagues PHN patients, so we included spontaneous pain in the index of observation.

**Figure 3:**
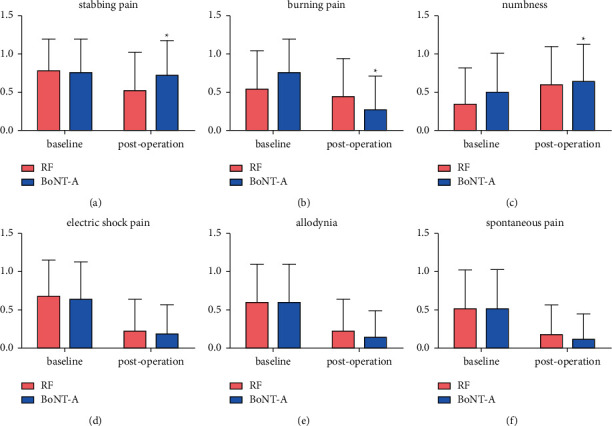
The incidence of different pain characteristics pre- and postoperation in two groups. (a) Stabbing pain, (b) burning pain, (c) numbness, (d) electric shock pain, (e) allodynia, (d) spontaneous pain. ^*∗*^=*P* < 0.05 vs. RF.

There was no difference in the incidence of different pain characteristics between the two groups at baseline, as shown in Table 1. At 24 weeks postoperation, the incidence of numbness in the two groups was increased compared with that at baseline, while the incidence of stabbing pain, burning pain, electric shock pain, allodynia, and spontaneous pain were decreased. Among them, the incidence of stabbing pain postoperation in the RF group was significantly lower than that in the BoNT-A group (*P* < 0.05), while the incidence of burning pain postoperation in the BoNT-A group was significantly lower than that in the RF group (*P* < 0.05), as shown in Figure 3.

### 3.5. Oral Analgesics

In this study, the oral analgesics included anticonvulsants (gabapentin), antidepressants (duloxetine), and opioids (tramadol or oxycodone). However, most patients resist taking antidepressants for long periods. Therefore, at the end of 24 weeks postoperation, we mainly observed two indicators: (1) the dose of gabapentin and (2) whether to take tramadol or oxycodone.

At 24 weeks after surgery, there was no difference in the dose of gabapentin between the two groups (*P* > 0.05), and there was no difference in the proportion of patients taking tramadol or oxycodone (*P* > 0.05), as shown in Table 5.

### 3.6. Costs

According to the national charging standard, the expenses of patients in the RF group (including surgery fee, material fee of puncture needles, and puncture guidance fee) were much higher than those in the BoNT-A group (including drug fee of botulinum toxin type A, and surgery fee) (*P* < 0.05), as shown in Table 5.

### 3.7. Adverse Event

Throughout the study, there was one adverse event in each group. One patient in the RF group developed pneumothorax after surgery. After bed rest, oxygen inhalation, and other treatments, the patient's pneumothorax was completely absorbed about a week later without any sequelae. One patient in the BoNT-A group, whose pain was located in the shoulder, developed upper limb lift weakness with grade 4 muscle strength on the second day after surgery. No special treatment was given. After 4 weeks, the patient's muscle strength was completely restored, and a sense of fatigue was reported. At the end of the 12th week, the fatigue of the patient disappeared completely. In general, both treatments are relatively safe.

## 4. Discussion

Postherpetic neuralgia (PHN) is the most common and disturbing clinical symptom of herpes zoster. The current clinical guidelines recommend pregabalin, gabapentin, tricyclic antidepressants, and 5% lidocaine patch as the first choice for the treatment of PHN [19, 22, 23]. For patients with severe pain, tramadol or strong opioid analgesics can be taken orally [19, 20]. For patients who do not respond well to oral medication, nerve block [24], nerve pulsed radiofrequency [9, 25], spinal cord electrical stimulation [26], etc. can be used as adjuvant treatments. However, these adjuvant treatments require skilled surgeons to perform with a precise positioning, and the operations are relatively complicated.

Considering the clinical efficacy, cost, and ease of use, the most commonly used adjuvant therapy for PHN is spinal nerve pulsed radiofrequency. Most patients with PHN experienced a significant reduction in pain after pulsed radiofrequency [9, 25]. However, pulsed radiofrequency requires precise positioning, and the operation is often dependent on the guidance of ultrasound or CT. Even so, some adverse events still occur from time to time, which is also happened in this study, as one patient in the RF group developed pneumothorax. Although it was discovered in time and healed quickly after treatment, it still caused a certain amount of trauma to the patient's body and mind. Additionally, the cost of pulsed radiofrequency is relatively high, and ultrasound or CT-guided puncture further increases the cost of treatment to some extent.

Although there are many drugs and methods to treat PHN clinically, there are still some refractory PHN, which does not respond well to various treatments, seriously affect the sleep quality and emotional state of patients, and may even cause anxiety and depression in patients. Pulsed radiofrequency technology by the needle will be intermittent (pulse frequency of 2 Hz), short-term (current role duration of 20 ms), frequency of 500 kHz high-frequency alternating current (ac) on the dorsal root ganglion, by adjusting the nerve function to achieve pain relief, with minimally invasive, safe, quick effect, less adverse reaction, etc. But its ability to relieve pain is limited. Kim et al. [27] applied X-ray-guided dorsal root ganglion pulsed radiofrequency technology to treat 29 PHN patients (disease course >3 months). Although postoperative pain was relieved to a certain extent, only 5 patients (17.2%) achieved more than 50% pain relief. Ding et al. [28] used CT-guided dorsal root ganglion pulsed radiofrequency technology to treat 50 PHN patients (course of disease >3 months), and only 14 cases (28%) achieved significant efficacy, namely, pain, numbness, and pain sensation disappeared, and labor ability returned to the level of onset. Wan et al. [29] reported that long duration and high voltage contributed to the improvement of pulsed radiofrequency efficacy. Although the effect of pulsed radiofrequency was improved by adjusting the treatment parameters, 46.2% patients still had no obvious or ineffective pain relief. In addition, even patients who achieve more than 50% pain relief are clinically observed to often have residual pain, especially away from the dorsal root ganglion.

Based on this, we found through the literature review that BoNT-A has certain therapeutic effects on PHN and can improve the quality of life of patients [12, 13]. Its mechanism of action may be related to SNAP-25 [30]. In vitro and in vivo studies have shown that BoNT-A suppresses the development of peripheral and central sensitization through reducing the pro-inflammatory and excitatory neurotransmitters and neuropeptides (such as substance P and calcitonin gene-related peptide) and glutamate release from primary afferent fibers [31–33]. BoNT-A also reduces the function of the pain-sensitive ion channels, such as transient receptor potential cation channel subfamily V member 1 (TRPV1), on the membranes of nociceptive neurons [34, 35]. However, previous studies have mostly used placebo as a control, which is somewhat different from actual clinical work. Therefore, we designed pulsed radiofrequency as a control to observe the efficacy and safety of subcutaneous injection of BoNT-A in the treatment of PHN.

Through the noninferiority study design, we found that BoNT-A was as effective in treating PHN as pulsed radiofrequency. Meanwhile, the patients in both groups were accompanied by a reduction in pain, a simultaneous improvement in sleep quality, and a significant reduction in anxiety and depression scores. Notably, the anxiety scores of the BoNT-A group were lower than that of the RF group at the 24 weeks postoperation. Overall, from the perspective of the three secondary outcome indicators of sleep quality, anxiety, and depression scores, the efficacy of BoNT-A is not inferior to that of pulsed radiofrequency.

In this study, we also noted that patients with PHN often complained of different pain characteristics. Therefore, we applied the five pain characteristics in the ID-Pain scale—stabbing pain, burning pain, numbness, electric shock pain, and allodynia, and then added the spontaneous pain to observe whether there were differences in efficacy for different pain characteristics between the two treatments. By comparison, the incidence of burning pain in the BoNT-A group was significantly lower than that in the RF group at the end of 24 weeks postoperation, while the incidence of stabbing pain in the RF group was significantly lower than that in the BoNT-A group. At the same time, except for numbness, the incidence of other pain characteristics in both groups was significantly lower than that at baseline. These results also indicate that BoNT-A can effectively treat PHN from another aspect.

Previous studies have suggested that different pain characteristics may implicate different mechanisms, including peripheral sensitization, central sensitization, inflammatory response, deafferentation, and ectopic pacemaker. [6, 36, 37]. Koltzenburg et al. reported that peripheral sensitization was mainly mediated by C fibers, and thermal hyperalgesia and static mechanical hyperalgesia could occur in the lesions [38, 39]. Simone et al. found that central sensitization could be caused by the pathophysiological changes, such as the up-regulation of central voltage-gated calcium channel *ɑ*2 − *δ* subunit and sodium channel, the decreased function of inhibitory neurons, and the necrosis of supporting cell [40]. The main clinical manifestations are spontaneous pain, allodynia, etc. [41]. With the degeneration and necrosis of primary afferent neurons, the excitability of secondary central neurons increased; that is, deafferentation, and pain and numbness may occur in the local lesions [42].

Limited by the small sample size, this study cannot precisely explain which treatment is more suitable for patients with specific types of painful PHN. To select more targeted treatments according to the pain characteristics of patients, future studies are required to understand the precise mechanisms underlying different pain characteristics.

## 5. Conclusions

Subcutaneous injection of BoNT-A can treat PHN and improve the quality of sleep and life effectively, with clinical efficacy comparable to pulsed radiofrequency. Although pulsed radiofrequency also showed excellent efficacy, it was more suitable for the treatment of large area PHN due to problems such as precise guidance and residual pain. For PHN patients with burning pain, BoNT-A can better improve the clinical symptoms of patients. In addition, the overall cost of subcutaneous injection of BoNT-A is relatively low, which is a relatively safe, effective, easy, and inexpensive method for the treatment of PHN.

## Figures and Tables

**Table 1:** Baseline information of the two groups of patients.

	RF	BoNT-A	*t*/*X*^2^/*Z*	*P*
Gender (male/female)	26/24	26/24	0	1
Age (Years)	72.28 ± 7.43	72.20 ± 6.57	0.057	0.955
Course^*∗*^	1(1–2)	1(1–2)	−0.110	0.912
NRS	6.00 ± 1.01	6.12 ± 1.10	−0.568	0.166
ID-pain	3(2–3.25)	3(3–4)	−0.828	0.408
ST (without/with)	11/39	12/38	0.056	0.812
BU (without/with)	23/27	21/29	0.162	0.687
NU (without/with)	33/17	25/25	2.627	0.105
EL (without/with)	16/34	18/32	0.178	0.673
AL (without/with)	20/30	20/30	0	1
SP (without/with)	24/26	24/26	0	1
SQS	8.70 ± 2.56	8.88 ± 2.65	−0.346	0.730
GAD-7	9.82 ± 3.96	10.24 ± 4.27	−5.10	0.611
PHQ-9	11.62 ± 4.24	12.28 ± 4.24	−0.778	0.439

^*∗*^The course of PHN was divided into 4 stages: 1 = ≤3 months, 2 = 3–6 months (including 6 months), 3 = 6–12 months (including 12 months), 4)≥c12 months.

**Table 2:** Five-item questionnaires and scoring system^*∗*^ used for the evaluation of quality of sleep.

	A	B	C	D
1. How would you describe the overall quality of your sleep during the last week?	Very bad	Rather bad	Rather good	Very good
2. How many nights during the last week did you feel you could not sleep because of pain?	7 nights	4–6 nights	1–3 nights	None
3. How many times was your night sleep interrupted because of pain during the last week?	≥5 times	3–4 times	1–2 times	None
4. How many hours of continuous night sleep did you experience during the last week?	1–2 h	3–4 h	5–6 h	7–8 h
5. How much time did you spend lying in bed before sleeping during the last week?	≥60 min	40–60 min	20–40 min	<20 min

^*∗*^Scoring system: A, 3 points; B, 2 points; C, 1 point; D, 0 point. Scoring deviation: 0 (min) to 15 (max), 0–5 points: good quality, 5–10: moderate quality, 10–15: bad quality.

**Table 3:** Comparison of GAD-7 scores at baseline and 24 weeks postoperation in both groups (*n* = 50, $\bar{\text{X}}\pm \text{S}$).

	RF	BoNT-A	*t*	*P*
Baseline	9.82 ± 3.96	10.24 ± 4.27	−0.51	0.647
24 weeks postoperation	5.94 ± 5.00	5.60 ± 4.22	0.368	0.039
*t*	4.309	5.46		
*P*	0.009	0.075		

**Table 4:** Comparison of PHQ-9 scores at baseline and 24 weeks postoperation in both groups (*n* = 50, $\bar{\text{X}}\pm \text{S}$).

	RF	BoNT-A	*t*	*P*
Baseline	11.62 ± 3.96	12.28 ± 4.24	−0.78	0.874
24 weeks postoperation	7.72 ± 5.00	8.16 ± 5.44	−0.04	0.416
*t*	3.94	4.22		
*P*	0.001	0.003		

**Table 5:** The oral analgesics of patients at 24 weeks postoperatively and the treatment-related costs during hospitalization (median and interquartile range).

	RF	BoNT-A	Z/*X*^2^	*P*
Gabapentin (g)	0.55 (0.45–0.74)	0.60 (0.44–0.68)	−0.374	0.708
Opioids	14 (28.0)	12 (24.0)	0.208	0.820
Costs (yuan)	6287.19 (6210.37–6415.53)	3374.86 (3289.15–4592.03)	−8.617	<0.05

## Data Availability

The data used to support the findings of the study can be obtained from the corresponding author upon request.
